# Towards Precise Interpretation of Oil Transformers via Novel Combined Techniques Based on DGA and Partial Discharge Sensors

**DOI:** 10.3390/s21062223

**Published:** 2021-03-22

**Authors:** Sayed A. Ward, Adel El-Faraskoury, Mohamed Badawi, Shimaa A. Ibrahim, Karar Mahmoud, Matti Lehtonen, Mohamed M. F. Darwish

**Affiliations:** 1 Faculty of Engineering, Delta University for Science and Technology, Mansoura 11152, Egypt; sayed.ward@feng.bu.edu.eg; 2 Department of Electrical Engineering, Shoubra Faculty of Engineering, Benha University, Cairo 11629, Egypt; mohamed.badawi@feng.bu.edu.eg; 3 Extra High Voltage Research Centre, Egyptian Electricity Holding Company, Cairo 11517, Egypt; dr.elfaraskoury21@gmail.com; 4 Department of Electrical Engineering and Automation, School of Electrical Engineering, Aalto University, 02150 Espoo, Finland or karar.alnagar@aswu.edu.eg (K.M.); matti.lehtonen@aalto.fi (M.L.); 5 Department of Electrical Engineering, Faculty of Engineering, Aswan University, Aswan 81542, Egypt

**Keywords:** dissolved gas analysis, partial discharge, PD sensor, power transformer, insulating oil

## Abstract

Power transformers are considered important and expensive items in electrical power networks. In this regard, the early discovery of potential faults in transformers considering datasets collected from diverse sensors can guarantee the continuous operation of electrical systems. Indeed, the discontinuity of these transformers is expensive and can lead to excessive economic losses for the power utilities. Dissolved gas analysis (DGA), as well as partial discharge (PD) tests considering different intelligent sensors for the measurement process, are used as diagnostic techniques for detecting the oil insulation level. This paper includes two parts; the first part is about the integration among the diagnosis results of recognized dissolved gas analysis techniques, in this part, the proposed techniques are classified into four techniques. The integration between the different DGA techniques not only improves the oil fault condition monitoring but also overcomes the individual weakness, and this positive feature is proved by using 532 samples from the Egyptian Electricity Transmission Company (EETC). The second part overview the experimental setup for (66/11.86 kV–40 MVA) power transformer which exists in the Egyptian Electricity Transmission Company (EETC), the first section in this part analyzes the dissolved gases concentricity for many samples, and the second section illustrates the measurement of PD particularly in this case study. The results demonstrate that precise interpretation of oil transformers can be provided to system operators, thanks to the combination of the most appropriate techniques.

## 1. Introduction

Dissolved gas analysis (DGA) is an efficient diagnostic way to the early detection of incipient transformer faults. Specifically, DGA is one of the most important tests for insulating fluid materials in the electrical components with different intelligent sensors. Besides, power transformers are considered the most key assets of electric power substations [1,2,3,4,5]. Practically, an oil transformer sample is regularly investigated at any time from all electrical devices without needing to turn it off. Key gasses formed by the degradation of oil and paper insulation are: (1) hydrogen (H_2_), (2) ethylene (C_2_H_4_), (3) acetylene (C_2_H_2_), (4) carbon dioxide (CO_2_), (5) ethane (C_2_H_6_), (6) methane (CH_4_), and (7) carbon monoxide (CO). The gas concentrations, generation rates, specific gas ratios, and the total combustible gas are important parameters for interpreting the result of DGA [6,7,8,9].

The six common faults type are partial discharge (PD), low discharge faults (D1), high discharge faults (D2), low thermal faults (T1) in oil or/and in a paper, below 300 °C, and above 300 °C of the paper has carbonized medium thermal faults (T2), high thermal fault (T3) of a temperature above 700 °C if there is strong evidence of carbonization of the oil. There are many traditional approaches for the transformer fault diagnosis by the DGA method which comprises the important gas analysis—e.g., Rogers Ratio technique, IEC gas ratio code (IEC 60599) [8], followed by Dornenburg Ratio technique, and Duval triangle technique. These traditional diagnostics approaches do not often yield accurate analysis while missing important incipient faults, thereby leading to the ‘no decision’ issue. To ensure great efficacy of accuracy and sufficient diagnostics of definite transformer fault types, numerous artificial intelligent techniques were executed. In turn, the artificial intelligence-based diagnostic techniques are an effective tool for maintenance transformer scheduling [10,11,12]. Hence, graphical DGA techniques are easy to be applied, nonetheless they still have limited diagnostic accuracies for different transformer faults [13,14]. More recently, the artificial neural network (ANN) is considered the most extensively used method in the literature for not only DGA but also diverse practical applications [15,16,17]. In [18], a fuzzy logic system is combined with a metaheuristic approach that is the hybrid grey wolf optimizer which adjusts DGA considering a diagnostic way that is robust against uncertainties. It is demonstrated that fuzzy logic, metaheuristics, and ANN can provide improved performance in general engineering applications [19,20,21,22,23]. Specifically in transformer oil diagnoses, [24] introduces an intelligent approach used to diagnose the fault and to make the decision for oil-immersed power transformers, based on the DGA principle. In [25], a deep parallel detection technique for dissolved gas analysis of the transformer is used. In [26], modified diagnosis techniques of fault types within oil-immersed power transformers using DGA and genetic algorithm software are presented.

As mentioned in the above literature, most of the existing DGA techniques can have poor diagnostic precision [27,28], while they may fail to interpret the oil faults in transformers. To solve this issue, this research work aims to fill this gap by improving the diagnostic accuracy of transformer faults. This ambitious task is accomplished by combining multiple techniques together in a unified framework, thereby contributing to maximize the diagnostic accuracy compared to individual approaches. Specifically, the first part is about the proposed techniques that are produced, and are classified into four techniques. These techniques were formed by combining the results of different conventional methods with methods from previous studies, as the results of these techniques improved compared to the methods involved in the formation of each technique separately. Technique no. 1 is constructed based on the outputs of three techniques, Duval, Roger’s four ratios refined, IEC refined techniques, while technique no. 2 is preceded based on three (DGA) methods (clustering, conditional probability, and Duval triangle). Technique no. 3 is depending on the diagnosis results of two techniques (the artificial neural network (ANN) technique and Roger’s refined method). Finally, technique no. 4 is depended on the combined results of techniques no. 2 and no. 3. The integration between these DGA techniques with partial discharge (PD) sensors overcomes the individual weakness and the differences between the conventional methods, and this is proved by using 532 samples from the Egyptian Electricity Transmission Company (EETC) substation. In addition, the second part of this research overviews the experimental setup for (66/11.86 kV–40 MVA) power transformer which exists in the EETC networks, the first section in this part illustrates the analysis of the dissolved gases concentricity in part per millions (ppm) for several samples, the second section in this part illustrates the measurement of partial discharge before and after oil terminated. The novelty of this paper is that the demonstration of four proposed techniques for transformer oil fault diagnosis. These constructions techniques are based on integration among the different DGA techniques. Also, it includes the dissolved gases analysis for many samples as well as a measurement of PD, particularly for (66/11.86 kV–40 MVA) power transformer.

## 2. Faults and Failure Mode in Transformers

The arrangement of the gas formed in a fault is specified by several factors. In addition, the specified gases which are realized in any gas sample noticed at the analysis are additionally influenced by features other than those linking to the fault. Fault gases are caused by partial discharge faults, arcing discharge faults, and thermal faults (hotspots) [29]. The generation of fault gases is strongly dependent on the temperature; from [30], it is demonstrated relative amounts of gas with approximate temperature. Specifically, hydrogen, as well as methane, start to form in minor amounts about 150 °C, H_2_ and C_2_H_4_ production go down as temperature rises. When the temperature reaches 250 °C, the production of ethane starts to be formed. In turn, the production of C_2_H_4_ begins at about 350 °C. Besides, C_2_H_2_ production starts between 500 °C and 700 °C. Greater amounts of C_2_H_2_ can only be formed above 700 °C by interior arcing. Starting at around 275 °C and on up, the making of ethane surpasses methane. At near 450 °C, hydrogen production surpasses all other gases until around 750 °C to 800 °C; formerly more acetylene is formed [30]. Recently, researchers have been prompted to direct their attention to biodegradable and renewable insulating materials for transformer oil [31].

### 2.1. Partial Discharge Fault

PDs look like short pulses that frequently goes together with the emission of sound, bright, heat, and chemical responses. The bases of partial discharges comprise voids and cracks in solid insulation, floating mechanisms—e.g., water drops—as well as air bubbles, and corona produced due to sharp ends of solid insulation, windings, or tank. After initializing, partial discharge can transmit with increasing intensity until releasing as an arc discharge. Typically, this type of fault is categorized by the production of H_2_ and CH_4_.

### 2.2. Arcing Discharge Fault

Arcing discharges produce excessive temperatures and high amounts of gases, mostly C_2_H_2_ and H_2_. This fault kind is very unsafe and if not managed, can reason extreme pressure in the transformer tank, triggering even a dangerous explosion.

### 2.3. Thermal Fault

Thermal transformer faults arise because of the hotness of conductors, short circuits, hotness of windings because of eddy currents, slack connections, and inadequate cooling. These faults can be categorized into (1) low-temperature fault for temperature up to 150 °C, (2) medium to high fault for temperature between 300 °C and 700 °C, and (3) high-temperature fault for temperature above 1000 °C. Furthermore, localized thermal faults are recognized as flashpoints. Thermal faults generation hydrocarbon gases, mainly C_2_H_4_, CH_4_, and C_2_H_6_. A thermal fault type at little temperature (<300 °C) products mostly methane and ethane and approximately ethylene. However, a thermal fault at advanced temperatures (>300 °C) products ethylene. The excessive the temperature becomes the better the production of ethylene.

## 3. Dissolved Gas Analysis: Interpretation Techniques

Several DGA techniques are used in the first part of this research and are briefly listed below.

### 3.1. Duval Technique

**Figure 1:**
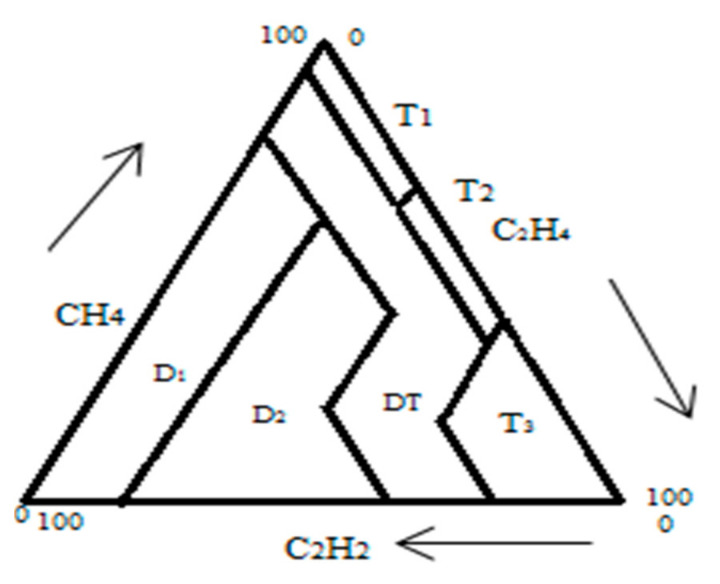
Description of Duval triangle.

This technique is built based on the attentiveness of three dissolved gases: C_2_H_2_, CH_4_, and C_2_H_4_. Usually, in the Duval triangle, seven zones are categorized as partial discharge (PD), thermal fault T1 (at T is less than 300 °C), thermal fault T2 (at T is between 300 °C and 700 °C), thermal fault T3 (at T is greater than 700 °C), low energy discharge (D1) sparking, high energy discharge (D2) arching and a maximum of thermal and electrical faults (DT). In order to diagnose the transformer fault using the Duval triangle (see Figure 1), the overall accumulated quantity of the three Duval triangle gases (C_2_H_2_, CH_4_, and C_2_H_4_) was considered and divided each gas by the total to find the percentage of each gas. Then, the fault type is determined by using the three ratios according to [32,33,34,35,36].

### 3.2. Roger’s Four Ratios Refined Technique

This technique is used to rise the correctness of Rogers’s four ratio method by adapting the ratio limits and their association between them to achieve the modified fault types link to the actual fault [37,38]. These four gas ratios are CH_4_/H_2_, C_2_H_6_/CH_4_, C_2_H_4_/C_2_H_6_, and C_2_H_2_/C_2_H_4_.

### 3.3. IEC Refined Technique

IEC refined technique is applied to raise the precision of the IEC method by adjusting the gas ratio limits and their connection between them to contract the correct fault types correspond to the actual fault [38,39]. The transformer faults type is determined by using three ratios CH_4_/H_2_, C_2_H_2_/C_2_H_4_, and C_2_H_4_/C_2_H_6_.

### 3.4. Conditional Probability Technique

Based on the percentage of the five main gases concerning their whole amounts, this method is utilized to diagnose transformer fault kinds. In this method, the probabilities of each fault type occurrence and non-occurrence are determined. Formerly, the probabilistic suggestion of each fault kind occurrence can be quantified according to the provisional probability of confident fault occurrence.

The transformer oil fault is classified as partial discharge (PD), arcing (AR), or thermal (TH), and the diagnostic is the fault type corresponding to the maximum probability [40,41].

### 3.5. Clustering Technique

This technique is based on the percentage of the five major dissolved gases—H_2_, C_2_H_2_, C_2_H_6_, C_2_H_4_, and CH_4_—with respect to their summation. The transformer fault is classified as partial discharge (PD), arcing (D1 and D2), or thermal (T1, T2, and T3). Based on the percentage ratio for each gas with respect to the sum of the five gases, the limit of each fault type in this method is determined [37,38].

### 3.6. Artificial Neural Network (ANN) Techniques

The ANNs are considered mathematical models that can be used in the modeling of composite structures. Specifically, ANNs contain three layers—the input layer, the hidden layer, and the output layer. The first layer characterizes the inputs while the third one models the outputs. The second layer contains nodes that attempt to functionally map the model inputs throughout optimization with respect to the model outputs [42,43].

Using the ANN technique, three DGA methods are combined. These methods are Rogers, IEC, and Duval. Each method consists of four-layer network (one input layer, two hidden layers, and one output layer). The input patterns are considered the gas ratios according to each method. Adopted weights represent the connection to the input while the product of weight and input yields the strength of it. A specified neuron receives multiple inputs from dissimilar sources. The output patterns are considered the main six fault types (PD, D1, D2, T1, T2, and T3) [44,45].

## 4. Proposed Techniques

Because of the importance of power transformers in the electrical grid and their high cost, it is necessary to develop fault diagnosis methods. DGA of different techniques is one of these methods used for the detection of transformer-oil faults. Therefore, new diagnostic methods should be developed based on the dissolved gases. Note that the construction of a large power transformer very depends on the current application or the type of the transformer, as in [46].

The proposed techniques are constructed based on the integration of the diagnosis results of recognized DGA techniques and are classified into four techniques.

### 4.1. Proposed Technique No. 1

This technique is constructed based on the outputs of three techniques, Duval, Roger’s four ratios refined, and IEC refined techniques. The inputs to this method are the combinations of outputs diagnoses from the three techniques. The integration between three DGA techniques is to improve the oil fault condition monitoring and overcome the individual weakness.

**Figure 2:**
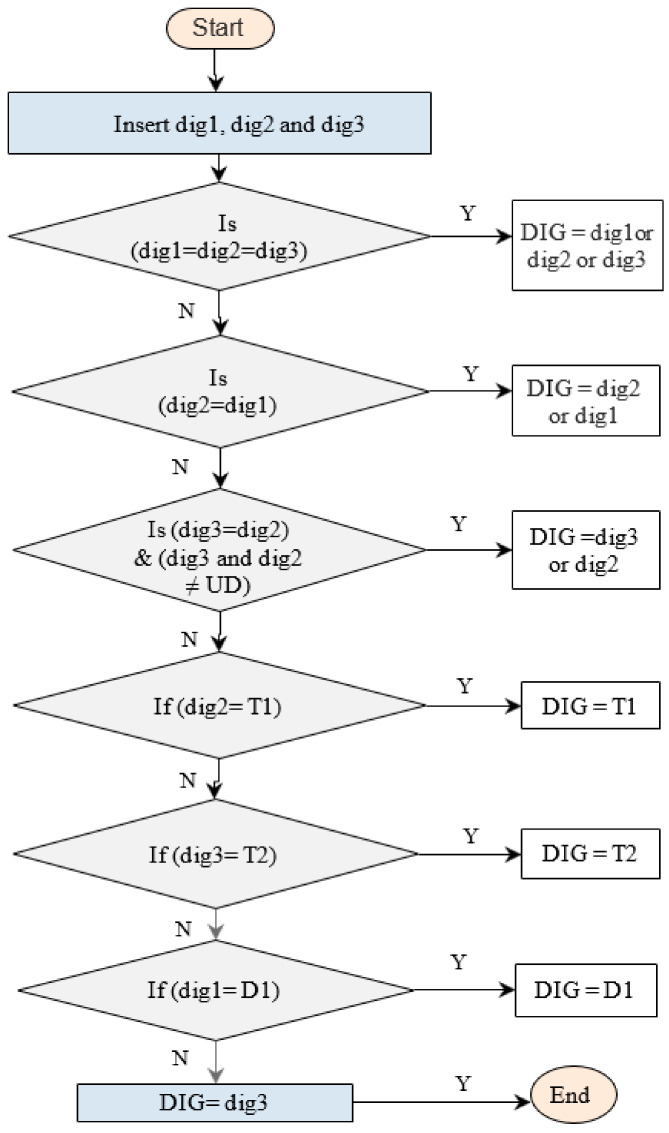
Flowchart procedure of technique no. 1.

Below, we describe the steps to apply this proposed combined technique (see Figure 2):

- When the three outputs (dig1, dig2, and dig3) are identical to each other, the diagnosis is one of these techniques;
- When the two outputs (dig2 and dig1) are identical to each other, the diagnosis is like dig2 or dig1;
- When the two outputs (dig2 and dig3) are identical to each other and are not equal to undetermined fault type, the diagnosis is like dig2 or dig3;
- When the diagnosis of dig2 is T1, the diagnosis is like dig2;
- When the diagnosis of dig3 is T2, the diagnosis is like dig3;
- When the diagnosis of dig1 is D1, the diagnosis is like dig1;
- When the three outputs (dig1, dig2, and dig3) are not identical to each other, the diagnosis is like dig3.

Where dig1, dig2, and dig3 are the diagnosis results by Duval and Rogers refined and IEC refined techniques, respectively.

### 4.2. Proposed Technique No. 2

This technique uses the integration among the outputs of three (DGA) techniques. These techniques are (clustering technique, the conditional probability technique, the Duval triangle method) [45]. This technique is associated with conventional methods represented by the Duval technique and unconventional methods represented by clustering and conditional probability techniques.

The steps of this technique are as follows:

- Insert the diagnostic outputs of the three above techniques (dig4, dig5, and dig1);
- If the three diagnostic outputs are identical, the diagnosis of the transformer fault type is identical to one of them (dig4 or dig5, or dig1);
- If the two diagnostic outputs (dig4 and dig5) are identical, the diagnosis of the transformer fault type is identical to one of them (dig4 or dig5);
- If the two diagnostic outputs (dig5 and dig1) are identical, the diagnosis of the transformer fault type is identical to one of them (dig5 or dig1);
- If the two diagnostic outputs (dig4 and dig1) are identical, the diagnosis of the transformer fault type is identical to one of them (dig4 or dig1);
- If the three diagnostic outputs are not identical, the diagnosis of the transformer fault type is dig5 due to higher accuracy compare to dig4 and dig1.

**Figure 3:**
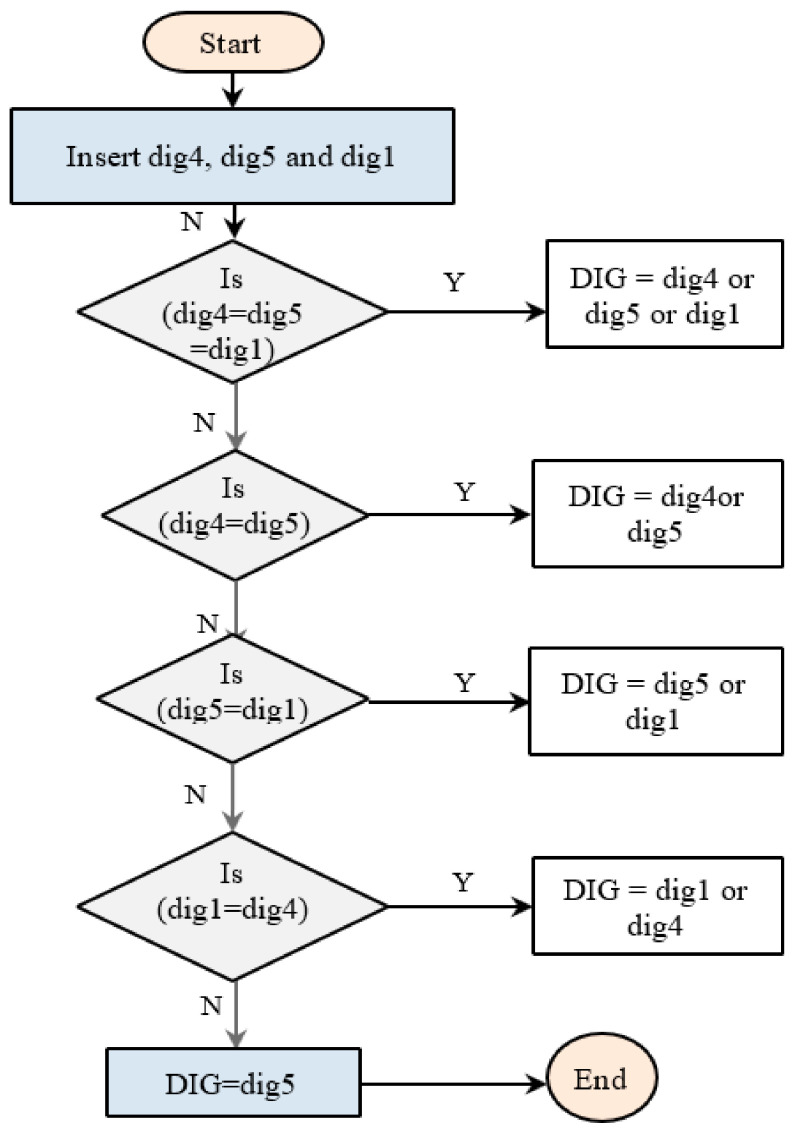
Flowchart procedure of technique no. 2.

Figure 3 illustrates the flowchart of technique no. 2 according to the above steps. Where dig1, dig4, and dig5 refer to the outputs of Duval triangle, clustering, and conditional probability techniques.

### 4.3. Proposed Technique No. 3

**Figure 4:**
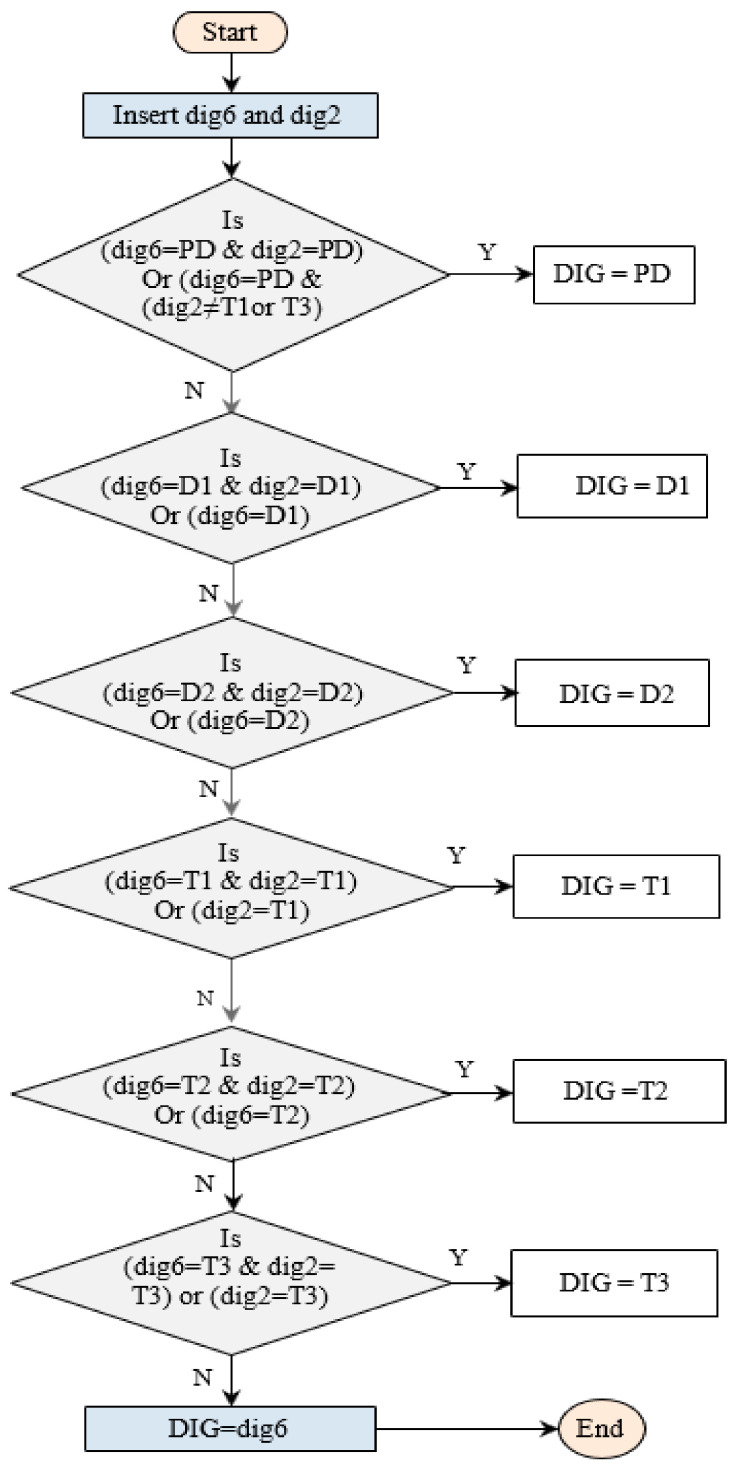
Flowchart procedure of technique no. 3.

This DGA technique is depending on the diagnosis results of two DGA techniques, the ANN technique and Roger’s refined method. This method links a conventional method with an unconventional method. The output of ANN’s technique and Roger’s refined technique (dig6, dig2) respectively are the inputs to the integrated technique (see Figure 4). The fault diagnosis using this DGA technique can be determined by following these steps:

- Insert the diagnostic outputs of the two techniques (dig6 and dig2);
- If the two diagnostic outputs are identical and give partial discharge fault type, or (dig6 diagnoses partial discharge fault type and dig2 is not diagnosed (T1 or T3)), the transformer fault type is partial discharge;
- If the two diagnostic outputs are identical and give low energy discharge (D1) fault type, or (dig6) diagnoses low energy discharge (D1) fault type, the transformer fault type is D1;
- If the two diagnostic outputs are identical and give high energy discharge (D2) fault type, or dig6 diagnoses high energy discharge (D2) fault type, the transformer fault type is D2;
- If the two diagnostic outputs are identical and give low thermal (T1) fault type, or dig2 diagnoses low thermal discharge (T1) fault type, the transformer fault type is T1.
- If the two diagnostic outputs are identical and give medium thermal (T2) fault type, or dig6 diagnoses medium thermal discharge (T2) fault type, the transformer fault type is T2;
- If the two diagnostic outputs are identical and give high thermal (T3) fault type, or dig2 diagnoses high thermal discharge (T3) fault type, the transformer fault type is T3;
- If the above steps were not achieved, the diagnosis of the transformer fault type is identical to dig6.

### 4.4. Proposed Technique No. 4

This technique uses the integration among the output results of the two techniques (no. 2 and no. 3) to improve the overall accuracy. During this technique construction, we take into account the merits and demerits of each of the two techniques (no. 2 and no. 3). The dig7 and dig8 refer to the results of technique no. 2 and technique no. 3 respectively and are the inputs to technique no. 4.

**Figure 5:**
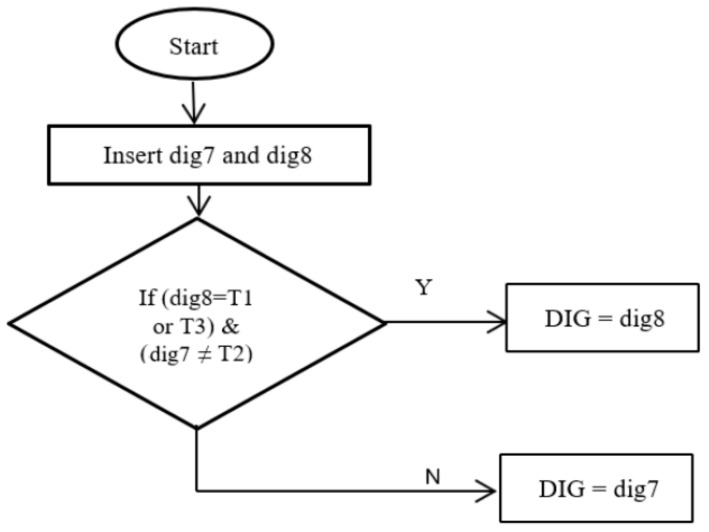
Flowchart procedure of technique no. 4.

The steps of this technique as follows: (see Figure 5)

- Insert the diagnostic outputs of the two techniques (dig7 and dig8).
- If the diagnostic output of dig8 is low or high thermal fault type and the diagnostic output of dig7 is not a medium thermal fault, the diagnostic fault is identical to the diagnostic output of dig8.
- If the above condition is not met, the diagnostic fault is identical to the diagnostic output of dig7.

## 5. Performance Evaluation of the Proposed Techniques

The training data set used was 532 samples from the Egyptian ministry of electricity, and were known for their actual faults (see Table 1). The performance of the proposed technique is evaluated by using MATLAB software.

**Figure 6:**
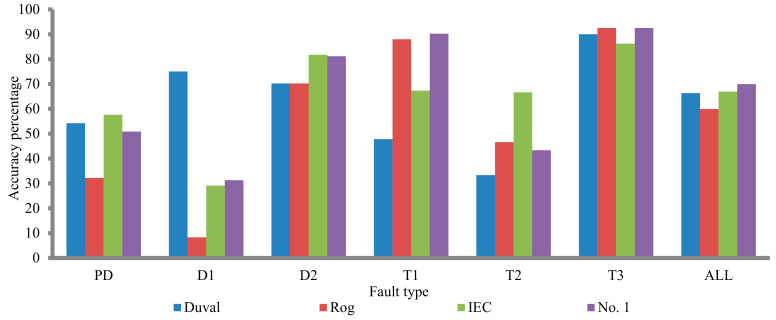
Comparison between the proposed technique no. 1 and its component method.

The total accuracy percentage of technique no. 1 is improved with compare to the three techniques (Duval triangle, Rogers refined, IEC refined) were produce this technique. The overall accuracy percentage of this technique is 69.92, whilst the total accuracy percentage of the three techniques (Duval, Rogers refined, and IEC refined) are 66.35, 59.9, and 66.9 respectively (see Table 2 and Figure 6).

The proposed technique no. 2 improved the overall accuracy percentage compare to the three techniques (Duval triangle, Conditional probability, and clustering techniques). The total accuracy percentage of this technique is 84.96, whilst the total accuracy percentage of the three techniques (Duval, conditional probability, and clustering approach) are 66.3, 82.8, and 62.7 respectively. The correctness of diagnosis D2 fault kind is enhanced from 70.2% for Duval and 92.5% for conditional probability and 94.2% for clustering method to 96% for technique no. 2, and the accuracy of diagnosis T1 fault type is improved from 47.8% for Duval and 82.6% for conditional probability and 29.3% for clustering method to 83.6% for the proposed method (see Table 3).

The total accuracy percentage of technique no. 3 is improved with comparison to the two techniques (ANN and Rogers refined four ratios) techniques. The overall accuracy percentage of it is 83.2, whilst the total accuracy percentage of the two techniques (ANN and Rogers refined four ratios) is 81.7 and 59.9, respectively. The accuracy of diagnosis T1 fault type is improved from 91.3 % for ANN and 88 % for Roger’s four ratios technique to 94.5% for technique no. 3 (see Table 4).

**Figure 7:**
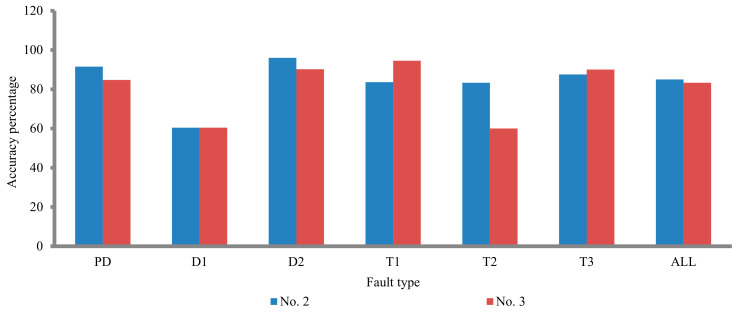
Comparison between the proposed techniques of no. 2 and no. 3.

By comparing the proposed techniques (no. 2 and no. 3), the detection of the fault types using no. 2 is better than of no. 3 except for T1 and T3 fault types (see Figure 7), so technique no. 4 is constructed to take into account the features of both techniques (see Table 5). The accuracy percentage is enhanced using technique no. 4 compare to the three techniques (no. 1, no. 2, and no. 3). The accuracy percentage of technique no. 4 is 85.3, whilst the accuracy percentage of the three techniques (no. 1, no. 2, and no. 3) is 69.92, 84.96, and 83.27, respectively, (see Table 6).

**Figure 8:**
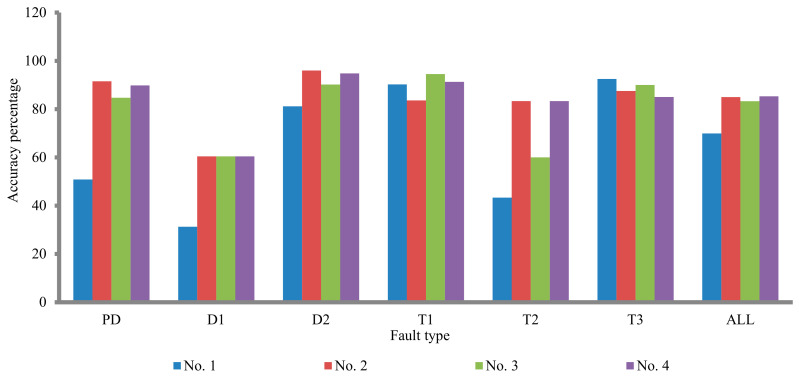
Comparison between the proposed DGA techniques.

- In the T3 fault type, the accuracy percentage of technique no. 1 is greater than the other techniques;
- In PD and D2 fault types, the accuracy percentage of technique no. 2 is greater than the other techniques;
- In the T1 fault type, the accuracy percentage of technique no. 3 is greater than the other techniques;
- In the D1 fault type, the accuracy percentage of technique no. 1 is less than the other techniques;
- In the T2 fault type, the accuracies percentage of techniques no. 1 and no. 3 are less than the two other techniques as shown in Figure 8.

## 6. Case Study

The (66/11.86 kV–40 MVA) power transformer has been analyzed and tested using DGA and partial discharge measurement [47,48,49].

### 6.1. Dissolved Gas Analysis (DGA)

The part per million (ppm) of the extracting gases were given for eight samples during the period from 8 November 2015 to 21 February 2019 as shown in Table 7.

**Figure 9:**
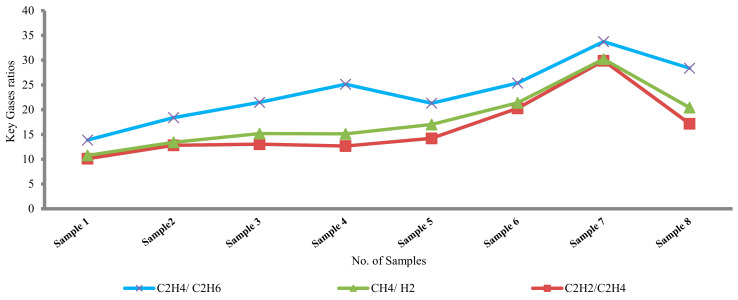
Analyzing the most common ratios in the case study.

The production of the most common gases (H_2_, CH_4_, C_2_H_6_, C_2_H_4_, and C_2_H_2_) by eight samples during this period is as Table 7. The concentrations of C_2_H_4_, C_2_H_6_, and following them (H_2_ and CH_4_) are the highest for eight samples. Figure 9 illustrates the production of the most common ratios (C_2_H_4_/C_2_H_6_, CH_4_/H_2_, and C_2_H_2_/C_2_H_4_). The ratio C_2_H_4_/C_2_H_6_ is higher than other ratios C_2_H_2_/C_2_H_4_ and CH_4_/H_2_. All proposed DGA techniques detected high thermal fault types for each sample such as laboratory results. After the oil has been purified, the transformer is returned to service.

### 6.2. By Using Partial Discharge Measurement

Based on the DGA of the case study, it showed an increase in the proportion of gases over the study period with high energy electrical arcing (the thermal fault of 700 °C), this means big eddy currents in tank and core, slight currents in tank walls caused by the excessive uncompensated magnetic field, shorted core laminations. This requires taking the decision to exit the network and conduct all tests with the recommendation to measure the percentage of partial discharge. Power transformers that are regarded as complying with IEC standards the same ways as new transformers shall be tested with 100% of the required test voltage. Transformers after many years in service or repair after breakdown shall be charged with 80–100% of the original test voltage.

(a) Induced Voltage Test with Partial Discharge Measurement (IVPD)

Induced voltage test with PD measurement according to IEC St. 60076-3 and IEC St. 60270 [36,37]. In the case of the test, frequency surpasses twice the nominal frequency, the computed time in seconds of the test will be (see Equation (1))

(1) $$Duration\;time=120\;\times \frac{rated\;frequency}{test\;frequency},but\;not\;less\;15$$

**Figure 10:**
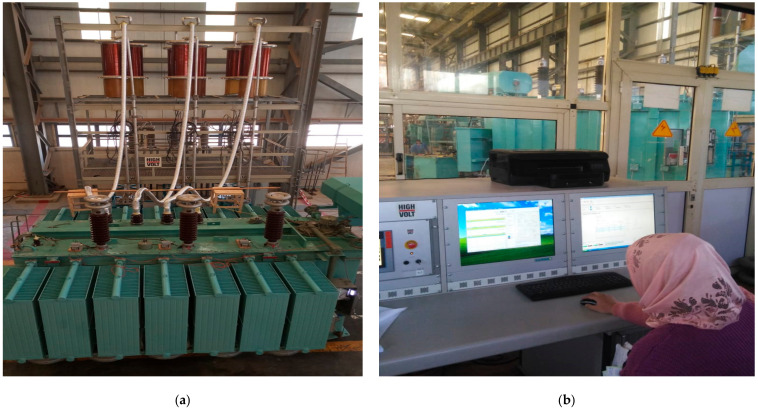
PD measurements; (**a**) power transformer under test, and (**b**) control system for the power transformer.

**Figure 11:**
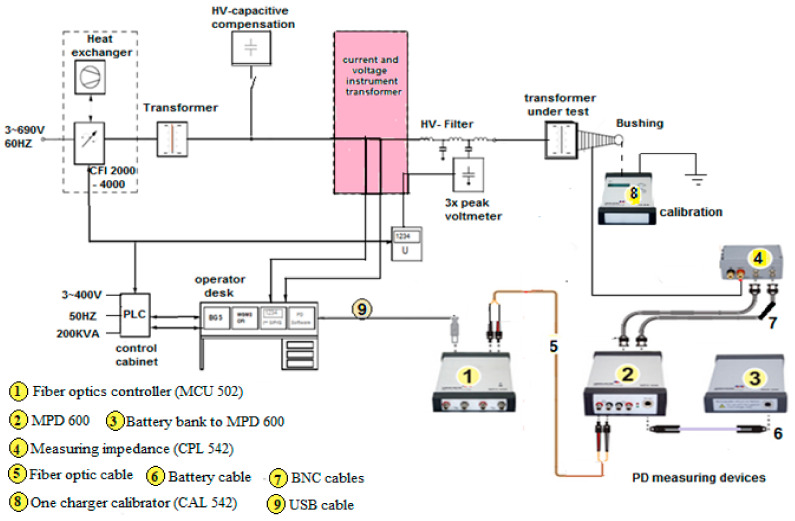
Partial discharge measurements experimental set-up.

In this case study, the rated frequency 50 Hz, the test frequency 150 Hz, and the duration time 40 s. The test shall begin at a voltage not higher than one-third of the quantified test value, and the voltage is enlarged to the test value as quickly as is reliable with practical tests. After the test, the voltage is minimized speedily to fewer than one-third of the test value beforehand switching off. However, the PD level can be unceasingly experiential on at least one measuring channel for the whole test period. Throughout the test sequence, the inception and extinction voltages of any important PD activity are required to be noted to aid the assessment of the test result if the test standards are not achieved [50,51,52]. Figure 10 shows the power transformer understudy and its control system using the condenser bushing test tap, where the capacitances between the measuring terminal and central conductor act as a capacitive voltage divider for the partial discharge signal. Furthermore, Figure 11 shows the online PD measurements experimental set-up with its components. 

**Figure 12:**
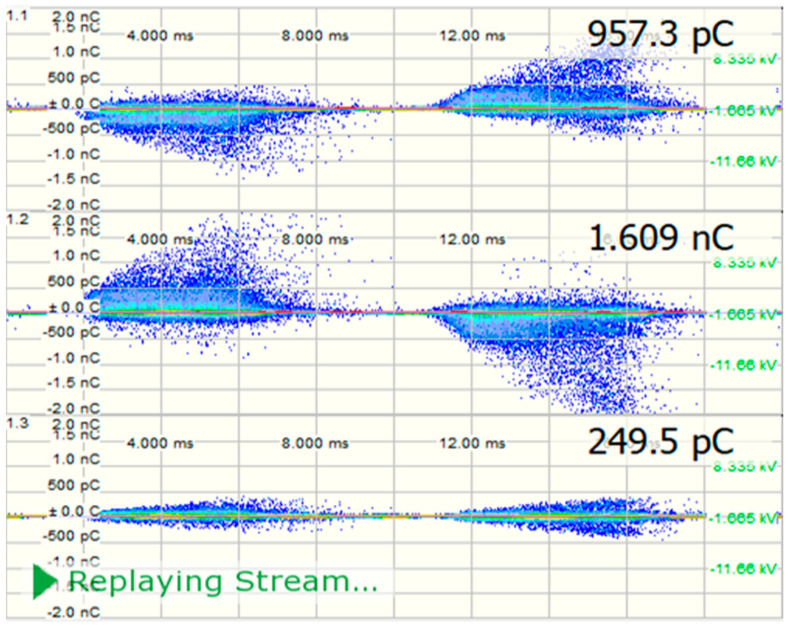
Synchronous multi-channel measurement and separation of PD.

**Figure 13:**
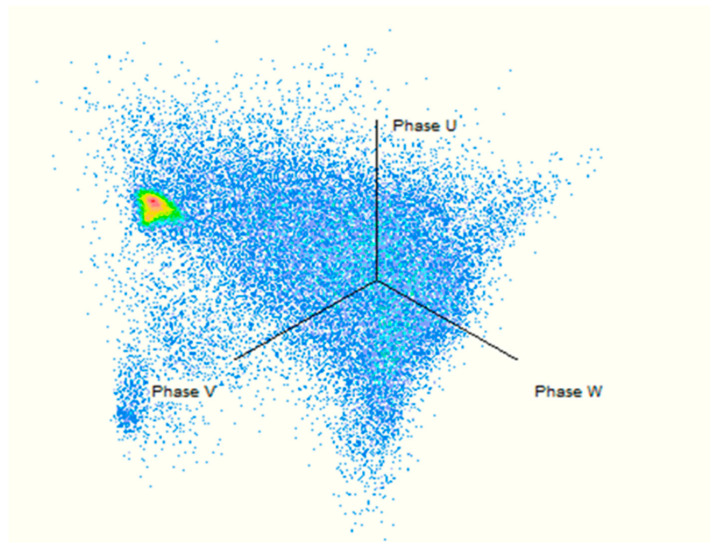
Different locations for PD (3PARD).

PD is measured through a technique according to IEC 60270 [49]. Every PD measurement channel includes the related bushing or coupling capacitor while calibrating in terms of apparent charge (pC). In this case, the PD device is connected to the bushing tap, if bushing tap connectors are not available, coupling capacitors are placed close to the bushing [53,54,55]. The PD measurement shall be assumed in pC and shall denote the maximum steady-state repetitive impulses specified by the measuring tool. The test can only be carefully effective if the measured background PD value does not surpass 50 pC at both the start and the termination of the test. For specified tests on shunt reactors, a background PD value of up to 100 pC may be accepted. By using OMICRON MPD 600 system and MI program, the results are obtained. Thence, Table 8 and Figure 12 and Figure 13 show the synchronous multichannel measurement and separation of PD and corona sources at different locations with three phase amplitude relation diagram (3PARD) before oil terminated.

The test proved the existence of partial discharge while the dissolved gas analysis did not refer to it, because the effect of high thermal fault type was stronger than it. Also, this test proved a defect for dissolved gas analysis, that only one fault type is diagnosed, which is the most effective.

**Figure 14:**
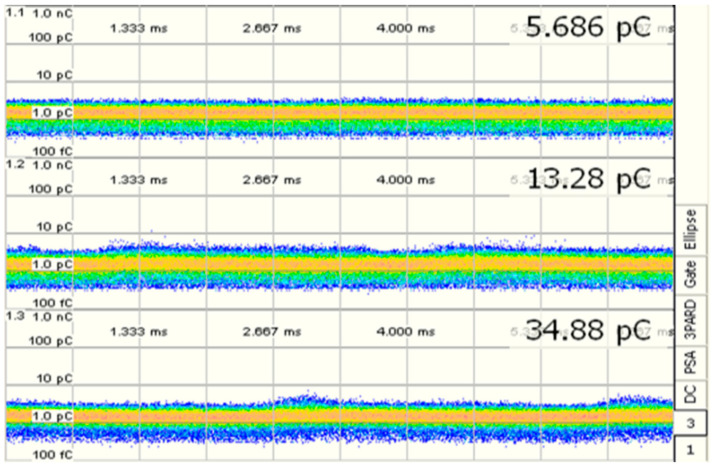
Synchronous multi-channel measurement and separation of PD After oil terminated (60 min).

**Figure 15:**
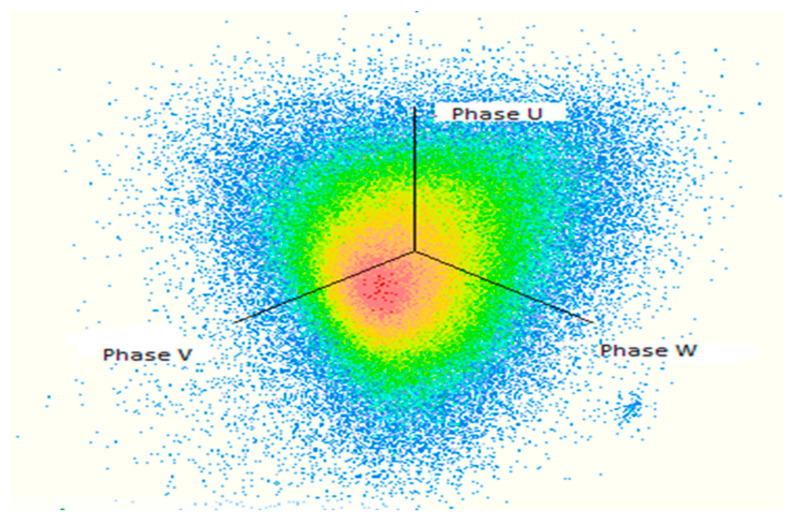
Different locations for PD with (3PARD) after oil terminated.

Since the presence of hydrogen and methane is related to the partial discharge failure, it must be taken into account if they are greater than or equal to 10 ppm, even if the other ratios are higher than them. Table 9 and Figure 14 show the results of PD measurements for power transformers after oil terminated overall period up to 1 h, and the different location for PD with three amplitude relation diagrams (3PARD) Shown in Figure 15.

**Figure 16:**
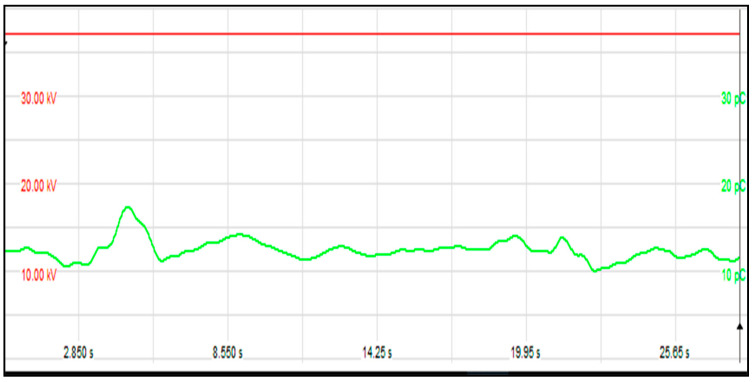
Relation between the raise of induced voltage and PD level.

Figure 16 shows an example of the relation between the raise of induced voltage at test frequency 150 Hz and the PD level during measurements for Phase V. The test is successful according to IEC Std. 60076 -3, where the PD value measured at a voltage value of (1.2 Ur)/√3 afterward the 1 h time does not surpass 100 pC, and the PD levels recorded during the one hour period exceed 250 pC, also the DGA after rapier and oil terminated within limit according to IEC Std. 60567 and IEEE Std. C57.104.

### 6.3. Discussion

By evaluating the results of the proposed technique:

- The diagnosis accuracy of the first technique is 69.92%. Furthermore, the accuracies of Duval, Rogers, and IEC techniques are 66.35%, 59.9%, and 66.9%, respectively.
- The diagnosis accuracy of the second DGA method is 84.96%. The accuracies of clustering, conditional probability, and Duval methods are 62.7%, 82.8%, and 66.3%, respectively.
- The diagnosis accuracy of the third DGA method is 83.2%. The accuracies of artificial neural network (ANN) and the Rogers methods are 81.7% and 59.9%, respectively.
- The fourth proposed method accuracy is higher than the accuracy of the first three methods.

According to the above section, the integration between DGA methods improves the accuracy of fault detection. This integration may be between conventional methods only or between conventional and unconventional methods or between unconventional methods only. For the case study of high thermal fault type according to DGA methods, the partial discharge levels are detected between phases U and V using electrical methods.

## 7. Conclusions

This paper introduces four techniques of DGA diagnosis for the interpretation of DGA results in mineral oil-filled transformers. These techniques constructions based on integration among the different DGA techniques. Also, it illustrates the dissolved gases analysis for many samples as well as a measurement of PD, particularly for (66/11.86 kV–40 MVA) power transformer. The first proposed technique is constructed based on the outputs of three techniques, Duval, Roger’s four ratios refined, IEC refined techniques, while the second technique is preceded based on three methods (new approach, conditional probability, and Duval triangle). The third technique is depending on the diagnosis results of two techniques (the artificial neural network technique and Roger’s refined method). The fourth proposed technique is depending on the results of the second and the third techniques. The first diagnosis technique has 69.92% accuracy as compared with 66.3% for Duval, 59.9% for Roger’s refined, and 66.9% for IEC refined. The second technique has 84.96% accuracy as compared with 66.3% for Duval, 82.8% for conditional probability, and 62.7% for clustering approach; the third technique has 83.27% accuracy as compared with 81.7% for ANN, 59.9% for Roger’s refined. The fourth proposed technique has 85.3% accuracy as compared with 84.96% for the second technique and 83.27% for the third technique. While the case study is analyzed using the dissolved for eight samples during the recent period, and the partial discharge is measured using electrical pulse detection. By analyzing the dissolved gases, the concentrations of C_2_H_4_, C_2_H_6_, and following them (H_2_ and CH_4_) are the highest for all samples, and the ratio C_2_H_4_/C2H_6_ is higher than other ratios C_2_H_2_/C_2_H_4_ and CH_4_/H_2_. The sensing of PD is measured experimentally at an induced voltage ratio of 1.58 Ur, and the partial discharge levels are detected between phases U and V. This case study summarized that we must not neglect hydrogen and methane if there are ppms that are greater than or equal to 10, even if the other ppms are higher than them. It also summarized the most important disadvantage of dissolved gas analysis, which is the diagnosis of only one fault type even if there is more than one fault. It should be taken into account in future research.

## Figures and Tables

**Table 1:** Number of samples for each fault type.

Fault Type	PD	D1	D2	T1	T2	T3	All
Sample	59	96	175	92	30	80	532

**Table 2:** Accuracies percentage of the no. 1, Duval, Roger’s four ratios refined and IEC refined methods.

Fault Type	Accuracy Percentage			
	Duval	Roger’s 4 Ratios Refined	IEC Refined	Proposed Technique No. 1
PD	54.2	32.2	57.6	50.84
D1	75	8.3	29.1	31.25
D2	70.2	70.2	81.7	81.14
T1	47.8	88.0	67.3	90.21
T2	33.3	46.6	66.6	43.33
T3	90	92.5	86.2	92.5
ALL	66.3	59.9	66.9	69.92

**Table 3:** Accuracy percentages of the modified technique and the three techniques.

Fault Type	Accuracy Percentage			
	Duval	Conditional Probability	Clustering Approach	Proposed Technique No. 2
PD	54.2	91.5	93.2	91.5
D1	75	61.4	35.4	60.4
D2	70.2	92.5	94.2	96
T1	47.8	82.6	29.3	83.6
T2	33.3	86.6	50	83.3
T3	90	80	47.5	87.5
ALL	66.3	82.8	62.7	84.96

**Table 4:** Accuracy percentages of no. 3, ANN, and Roger’s 4 ratios refined techniques.

Fault Type	Accuracy Percentage		
	ANN	Roger’s 4 Ratios Refined	Proposed Technique No. 3
PD	84.7	32.2	84.7
D1	60.4	8.3	60.4
D2	90.2	70.2	90.2
T1	91.3	88.0	94.5
T2	60	46.6	60
T3	83.7	92.5	90
ALL	81.7	59.9	83.27

**Table 5:** Accuracy percentages of no. 2, no. 3, and no. 4 techniques.

Fault Type	Accuracy Percentage		
	Proposed Technique No. 2	Proposed Technique No. 3	Proposed Technique No. 4
PD	91.5	84.7	89.8
D1	60.4	60.4	60.4
D2	96	90.2	94.8
T1	83.6	94.5	91.3
T2	83.3	60	83.3
T3	87.5	90	85
ALL	84.96	83.27	85.3

**Table 6:** Accuracy percentages of the four proposed techniques.

Fault Type	Accuracy Percentage			
	No. 1	No. 2	No. 3	No. 4
PD	50.84	91.5	84.7	89.8
D1	31.25	60.4	60.4	60.4
D2	81.14	96	90.2	94.8
T1	90.21	83.6	94.5	91.3
T2	43.33	83.3	60	83.3
T3	92.5	87.5	90	85
ALL	69.92	84.96	83.27	85.3

**Table 7:** DGA history of the case study.

The Date	H_2_	CH_4_	C_2_H_6_	C_2_H_4_	C_2_H_2_
Sample 1	18	12	136	421	0
Sample 2	18	11	95	472	7
Sample 3	38	81	57	359	3
Sample 4	38	93	62	621	1
Sample 5	48	134	131	561	3
Sample 6	31	34	177	706	9
Sample 7	28	9	126	444	6
Sample 8	36	117	89	707	7

**Table 8:** Induced voltage test with PD measurement (IVPD) before oil terminated.

Induced Voltage (kV)—150 Hz			Time	PD Level (pC)		
Ratio	LV Ph-Ph	HV Ph-Ph		Ph(U)	Ph(V)	Ph(W)
1.8 Ur	21.34	118.8	00:00:40	-	-	-
1.58 Ur	18.73	104.2	00:60:00	957.3	1609	249.5

**Table 9:** Induced voltage test with PD measurement (IVPD), after oil terminated.

Induced Voltage (kV)—150 Hz			Time	PD Level (pC)		
Ratio	LV Ph-Ph	HV Ph-Ph		Ph(U)	Ph(V)	Ph(W)
1.8 Ur	21.34	118.8	00:00:40	-	-	-
1.58 Ur	18.73	104.2	00:60:00	5.686	13.28	34.88
1.58 Ur	18.73	104.2	00:55:00	8.232	15.63	54.42
1.58 Ur	18.73	104.2	00:50:00	7.66	14.92	49.15
1.58 Ur	18.73	104.2	00:45:00	7.11	14.45	43.30
1.58 Ur	18.73	104.2	00:40:00	6.73	13.31	34.53
1.58 Ur	18.73	104.2	00:35:00	8.002	23.06	32.93
1.58 Ur	18.73	104.2	00:30:00	10.27	35.81	27.55
1.58 Ur	18.73	104.2	00:25:00	5.03	12.60	19.80
1.58 Ur	18.73	104.2	00:20:00	4.89	12.38	24.62
1.58 Ur	18.73	104.2	00:15:00	4.85	12.69	17.64
1.58 Ur	18.73	104.2	00:10:00	5.07	12.94	20.42
1.58 Ur	18.73	104.2	00:05:00	5.57	12.84	18.43

## Data Availability

The data presented in this study are available on request from the corresponding author.
